# Experiences of interpersonal victimization and abuse among autistic people

**DOI:** 10.1177/13623613231205630

**Published:** 2023-10-16

**Authors:** Sarah Douglas, Felicity Sedgewick

**Affiliations:** University of Bristol, UK

**Keywords:** autism, intimate partner violence, relationships, sexual assault

## Abstract

Intimate partner violence and sexual assault are under-researched experiences in autistic people’s lives. Recent research, however, has shown that autistic people are more likely to have been victimized than non-autistic people. This research, therefore, sought to explore the firsthand accounts of a range of autistic people about intimate partner violence and sexual assault. Twenty-four autistic adults with lived experience (6 male, 15 female, 3 non-binary) aged 25–61 years took part in semi-structured interviews online. They were asked about their experiences of intimate partner violence and sexual assault, whether and how they felt being autistic interacted with those experiences, and what recommendations they would have for improving education in the future. Almost all participants had repeated experiences of intimate partner violence and sexual assault, regardless of gender, and there were clear similarities in their stories. Six themes with subthemes were identified. These were ‘*experiences of abuse*’, ‘*autism used against you*’, ‘*poor family models*’, ‘*impact of/on friendships*’, ‘*handling trauma*’, and ‘*recommendations for future practice*’. Autistic people experience many of the same patterns of abuse as non-autistic people do, but there are unique autism-related vulnerabilities and outcomes. We found that there were a variety of responses to these experiences, and call for greater understanding so that autistic victims can be better supported.

**Lay abstract:**

**What do we already know?**

Autistic people are more likely to have negative life experiences than non-autistic people, from bullying and ostracization, to being victims of crime, to unemployment and homelessness. This includes being victims of intimate partner violence, sexual assault and domestic abuse. Quantitative work has suggested that as many as 90% of autistic people experience these forms of abuse in some form during their lives, but there is little work asking them to talk about harmful relationships in their own words.

**What does this article add?**

This article reports on interviews with 24 autistic adults about their experiences of being victims of intimate partner violence, sexual assault and/or domestic abuse. Some of the themes which came from these interviews are shared with non-autistic victims, but others appeared unique to autistic people. One of these was evidence for unique autism-related vulnerabilities, as well as the impact the abuse had on their relationships long term. Participants also talked about how the sex and relationship education they had received had inadequately prepared them for adult relationships, and how this had contributed to their struggle to recognize and react to abusive behaviour.

**Implications for practice, research and policy**

Policies around intimate partner violence and sexual assault need to be updated to account for the different ways in which neurodivergent people (people whose brains process information differently from the majority) may discuss their experiences, rather than looking for ‘standard narratives’ as an indicator of a need for support. Relationship and sex education should be tailored for autistic young people to help them recognize abusive behaviours, and include how to respond to these safely. We recommend that future research tries to focus specifically on the abuse experiences of autistic men, non-binary and trans people, who have been under-represented in studies to date. In addition, much less is known about the abuse experiences of autistic people of colour or autistic people with intellectual disabilities, who also need to be actively included in these discussions.

## Introduction

Autism is a neurodevelopmental condition associated with differences in social interaction, executive function, repetitive behaviours (also known as self-stimulating or stimming) and intense interests, and sensory sensitivities (American Psychiatric Association, 2013). Around 1%–2% of the population are autistic, with a gender disparity in diagnosis rates – around three males are diagnosed for every one female (Loomes et al., 2017), with an increasingly recognized pattern of under- or mis-diagnosis for women (Gould, 2017). Historically, research into autistic experiences has focused on males, and particularly on children, meaning that the understanding of adult life experiences, such as romantic or sexual relationships, and especially those of women and non-binary people, is severely lacking. For a long time, it was assumed that most autistic adults did not often have romantic or sexual relationships, and indeed that generally they would not live independently (Howlin, 2000). While there have long been a wealth of first-person accounts challenging this stereotype, it is only recently that formal research has started to investigate the sexual and romantic experiences of autistic adults.

The first thing to note is that autistic adults can and do have sexual and romantic relationships, and that while there is some evidence that their relationships may tend to be of shorter duration (Hancock et al., 2020), this is not universal, and autistic people can be in happy partnerships for decades, just as non-autistic people can. Indeed, one recent study has suggested that neurodivergent people (people whose brains process information differently from the majority), – in this case, specifically those who were autistic or had attention deficit hyperactive disorder (ADHD) – may show more passionate love in their relationships, rather than less (Soares et al., 2021). Around half of autistic adults without learning difficulties are in long-term relationships, mostly living with their partner (Dewinter et al., 2017), and when asked about the quality of those relationships, they rate them just as close, supportive and important as non-autistic people do (Sedgewick, Leppanen, & Tchanturia, 2019).

Unfortunately, what we also know is that autistic adults often experience some form of intimate partner violence (IPV) or sexual abuse (SA),more so than non-autistic adults. On a measure of general negative life experiences, including financial hardship, homelessness and domestic abuse, autistic adults were several times more likely to have been in these situations (Griffiths et al., 2019). A further meta-analysis looking at the prevalence of victimization among autistic people showed that 40% of people reported sexual victimization, 16% reported being abused as a child and 84% of autistic adults reported having been victimized in more than one of the potential categories (Trundle et al., 2023). These are stark statistics – it means that the majority of autistic adults have experienced at least one period of victimization, and most will have been victimized multiple times in their lives, often by people who they should have been able to trust such as friends, family members or carers. This appears to be particularly the case for autistic women and people assigned female at birth, with between 60% and 90% reporting some form of serious sexual assault or domestic violence experience (Cazalis et al., 2022). Saying that, there is some evidence that autistic men, non-binary and trans people also experience elevated rates of unwanted sexual contact (Brown et al., 2017; Brown-Lavoie et al., 2014). For example, sexual victimization rates have been shown to range from 27% for cisgender men to as high as 85% for trans men (Reuben et al., 2021), showing that this is an important issue for autistic adults of all genders.

The above articles, and much of the work on autistic experiences of abuse and victimization in general, have been quantitative, attempting to gain an accurate picture of the scale of the problem. Less work has been done using a qualitative approach, where autistic people are asked to share their experiences in their own words. One study which has done this, however, interviewed more than 100 autistic people about having been victimized by people in their lives such as family members, friends and romantic or sexual partners (Pearson et al., 2023). Starting in childhood, participants shared examples of times they had been hurt, manipulated and abused by these people, and how this formed their expectations for relationships in general.

Another small-scale study into the romantic relationships of autistic women found similar patterns, with most having been victimized at some point, often repeatedly (Sedgewick, Crane, et al., 2019). In reflecting on these experiences, women also discussed how they felt being autistic had interacted with the malicious intent of their abusers to make them less able to recognize when something was unusual (even if they knew that they personally did not like what was happening), to communicate their lack of consent or desire to leave a situation, or to see the hidden messages in other people’s behaviours. Combined with having fewer friends they could talk to about their experiences and check whether the treatment from the abuser was normal, they often ended up staying in abusive relationships longer than they felt they should have, because they lacked the social support to recognize domestic violence and then to end the relationship – a crucial factor in leaving safely.

Participants in these studies especially emphasized how difficult it was for them to recognize that what had happened was wrong, and that trying to find ways to socialize safely with a background model of relationships as hurtful was exhausting, difficult and could lead to overwhelm and burnout. These negative impacts of the abuse they had experienced lasted well past the point of initial harm, with many developing post-traumatic stress disorder (PTSD). This is something we see in non-autistic victims as well (Dutton et al., 2006), and it is not surprising considering that autistic people are generally more likely to develop PTSD (Rumball et al., 2020), and to suppress their trauma-related thoughts which can lead to longer lasting harm (Golan et al., 2022). Indeed, the events which lead to PTSD have been shown to differ for autistic and non-autistic people, with autistic people being much more likely to have been traumatized by a social event (Haruvi-Lamdan et al., 2020), which would include IPV or SA. It is worth noting, though, that autistic women have talked previously about having found ways to thrive and be successful despite the trauma they have been through and the impact it has on them (Webster & Garvis, 2017), something which has not been thoroughly explored.

The current study sought to expand the body of literature qualitatively exploring autistic people’s experiences of IPV and SA, explicitly including autistic people of all genders. The limited work in this field to date means that much is still to be learned and understood about how autistic people understand, manage and recover from these kinds of trauma. Improving knowledge about these topics in a more diverse group of autistic people can help to inform education and support practices. We sought to discover whether there are common patterns in experiences across genders; the ways in which autistic people feel being autistic impacts their experiences; and what they felt could have helped them prepare for entering romantic and sexual relationships more safely.

## Methods

### Participants

Twenty-four autistic adults (6 cisgender male, 15 cisgender female, 3 non-binary) participated in semi-structured interviews (Mean age = 39.15, SD = 11.82, range: 25–61). All but one participant reported having formal autism diagnoses, from a range of practitioners, with one person being self-identified (Mean age at diagnosis = 31.83, SD = 15.22, range: 8–59). All participants completed the Autism Quotient – 10 item version (AQ-10; Allison et al., 2012) to enable description of autism symptomatology in a brief manner. Only one non-binary participant scored below the recommended cut-off point, but was retained in the sample due to recognized biases in the ability of the AQ-10 to identify autistic non-males (Mean score = 8.17, SD = 3.15, range: 4–10). Twenty-one participants reported additional physical and mental health diagnoses, with anxiety (n = 11), PTSD (n = 4) and depression (n = 7) being the most common. Sixteen participants reported having more than one additional physical and mental health diagnosis, and one participant reported co-occurring ADHD. Twenty-three participants identified as White (from the United Kingdom, United States and Australia), with one person identifying as Latino (from the United States). Twenty-one participants were based in the United Kingdom, two in the United States and one in Australia. Specific information on sexuality, socioeconomic status and educational attainment were not collected.

Recruitment took place via advertising the study on social media (Twitter, Facebook), and through the researcher’s personal networks, calling for participants who were autistic and had lived experience of IPV/SA. They were informed that this was an interview-based study about their experiences, and that they had the option of communicating via their preferred method, as well as being offered the chance to see the planned interview questions in advance of taking part. Ethical approval was granted by the University of Bristol School of Education’s Ethics Committee, and participants provided written informed consent before taking part, along with confirmatory verbal or written consent prior to beginning the interview. The sample is majority non-male, which differs to the general diagnosis pattern for autism, which stands at 3:1 male: female (Loomes et al., 2017). While this means the sample is not necessarily representative of all autistic people, it is not unexpected because the majority of victims of IPV and SA are female, and there is no reason to assume that would differ for the autistic population.

### Materials

#### Demographics

Participants verbally completed a short demographics questionnaire before the semi-structured interview began, answering questions about their age, gender, ethnicity, autism diagnosis and additional diagnoses.

#### Autism quotient – 10 item version (AQ-10)

Participants verbally completed this measure (Allison et al., 2012). The questionnaire assesses levels of autistic traits and consists of 10 statements. Participants answered ‘Yes’ or ‘No’ (adapted for simplicity) as to whether they identify with each statement. Scores range from 0–10, with a cut-off score of six or above being considered to reflect potentially clinical levels of autistic traits.

#### Semi-structured interview

The interview schedule was co-produced between the two authors, one of whom is multiply neurodivergent, including being autistic. Questions covered experiences of IPV and sexual victimization; whether and in what way participants felt being autistic interacted with these experiences; the sex and relationships education they had received; and what they thought could be done to improve this education. The full interview schedule can be found in Supplementary Materials.

### Procedure

Data were collected between May and August 2020, during the early phase of the COVID-19 pandemic. Due to this global context, all interviews were conducted online. Participants were offered the choice of video on (n = 20), video off (n = 1) or to communicate through typed chat (n = 3). Non-typed interviews were audio recorded and transcribed verbatim. Participants each received a £20 Amazon voucher as a thank-you for taking part in the study.

### Analysis

Transcripts were subject to inductive thematic analysis, that is, without any theoretically grounded, predetermined themes and as far as is possible, autonomous of the researcher’s preconceptions. This process followed the six steps outlined by Braun and Clarke (2006). The first three stages were conducted independently by the two authors: (1) data familiarization, (2) generation of initial codes from semantic content, (3) searching for themes which can be descriptive overviews of those codes. The two authors then met multiple times to proceed through the final three stages: (4) reviewing themes through discussion between the authors, (5) agreeing on themes and subthemes, and naming them and (6) report production. There was a high level of agreement on the initial codes the authors identified, though this was not quantified, with initial differences in how these were grouped into themes. These independent themes were refined through discussion into those presented in this article.

### Community involvement statement

From the start, this has been a co-produced project. The research team are neurodiverse, with personal experience of the issues covered, as well as experience in counselling neurodivergent survivors of abuse. Lived experience has therefore informed every stage of this project, from conception and interview design, to manuscript production.

## Results

**Figure 1.**
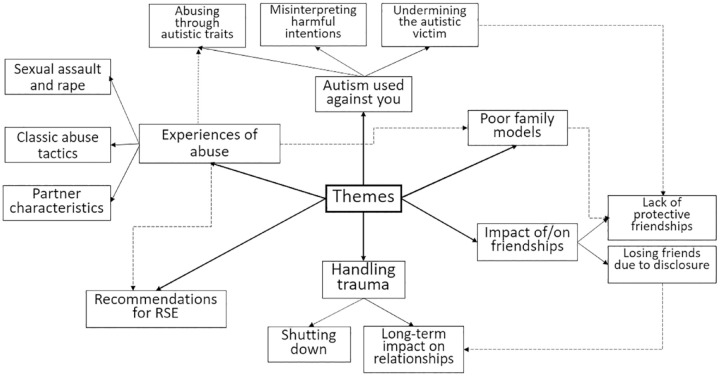
Thematic map. Solid arrows represent direct theme/subtheme relationships; dashed arrows represent connections between separate themes and subthemes. RSE: relationship and sex education.

Six themes were identified in the data, with additional subthemes. These are visualized in Figure 1. Solid arrows denote connections between themes and their subthemes, dashed arrows denote connections between separate themes and subthemes. Please note that due to some potential participants withdrawing between registration and taking part in the interview, participant numbers go to 29 although 24 people were included in the final dataset.

### Experiences of abuse

There were striking similarities in the stories of our participants – although the specific ways in which they had been abused may have been different, they described similar patterns of prevalence of abuse, persistence of abuse, features of their abusers and abusive relationships having been a recurring pattern in their lives.

#### Sexual assault and rape

The majority of our female and assigned-female-at-birth participants had been physically sexually assaulted or raped, both by strangers and by friends or acquaintances, as had some cisgender male participants. This form of victimization was something that they had encountered within multiple types of interpersonal contexts, both inside established abusive relationships and by people who they had no previous interactions with:

*I was raped when I was 17 . . . [by] a friend of a friend, somebody I knew . . . and it’s sort of happened a couple more times since then. One time I was drugged in a nightclub. (Participant 4, autistic woman)*

*I also was assaulted by a random stranger once on a bus . . . he started putting his hands under my clothes and he tried to get me to put my hand on his penis. (Participant 8, autistic woman)*

*I was raped by an older boy in primary school . . . and I was sexually assaulted by a teacher in secondary school. (Participant 16, autistic man)*

In addition, participants of all genders talked about having been coerced into having sex or specific sexual activities by their partners:

*if I like, just wasn’t in the mood, she would make me feel really guilty and say that it meant I wasn’t attracted to her, that I didn’t love her. (Participant 2, autistic non-binary person)*

*You do actually get to the point where you just think ‘Actually, if I fight this, it’s going to be hell anyway, one way or the other’. We might as well get [it over with.] (Participant 17, autistic woman)*

#### Classic abuse tactics

In addition to sexual assault, which was as often reported being committed by strangers as established partners, tactics which abusers used against the autistic people in this study also aligned with the commonly recognized behaviours in abusive relationships. Examples such as gaslighting (psychologically manipulating someone into doubting their own recollections of events or their mental health), coercion, physical, financial abuse and isolation are outlined below:

***Gaslighting***: *‘the guy actually said to me it’s all in my head, you know, it didn’t really happen, so it’s actually made me think not to say anything in the future’. (Participant 14, autistic woman)*

***Coercion***: *‘being pressured into doing stuff that I wouldn’t normally do to get a job and stuff like that’. (Participant 4, autistic woman)*

***Physical abuse***: *‘he headbutted me and left a goose-egg on my forehead’. (Participant 3, autistic woman)*

***Financial abuse***: *‘what I hadn’t realised is that she’d been siphoning money off to herself . . . put [joint savings] into her own account’. (Participant 15, autistic man)*

***Isolation*:** *‘she told all her friends I was abusive . . . she spent a lot of time making sure she spoke to the right people about how horrible I was . . . I got pushed out, especially in our church’. (Participant 12, autistic man)*

Equally, other people found that abusers tried to use systems of power to continue to hurt them or impact their lives, such as blocking a path to university (a physically and sexually abusive father exercising coercive control), trying to take more than what was fair in a divorce (an abusive ex-husband) or weaponizing the legal system to continue sending abusive messages:

*initially there was a refusal of contact with the child which we had to go to court and get resolved, we now have the Court Order . . . there are repeated calls to Social Services with made up allegation’. (Participant 15, autistic man)*

*I still have to be in contact with him [for the kids], he still is abusive over text message. (Participant 3, autistic woman)*

#### Partner characteristics

There was a significant diversity among the perpetrators of abuse against our participants, including family members (in childhood), teachers (also in childhood), friends or peers (at school or university), opposite- and same-sex partners and strangers. For those who were recounting abuse at the hands of romantic partners, a shared pattern was that those partners were also neurodivergent in some way, had mental health issues or had traumatic life histories:

*he was autistic too, and would say that what he did was my fault because I was triggering him, his sensory stuff, or because I was being unreasonable in asking for my accommodations so he had to do these things to show me how to properly be his partner. (Participant 17, autistic woman)*

*she had her own emotional issues, she was a narcissist . . . she came from a very controlling environment and learned it all from her mother. (Participant 12, autistic man)*

These characteristics were then seen as underlying causes for the abusive behaviour of their partners, and so the autistic victims spent a lot of time rationalising how they had been treated:

*he did get a diagnosis of BPD (Borderline Personality Disorder) . . . he was medicated for a little bit and the abuse stuff got better, but he got allergic to the medication, and that’s when the final break happened ‘cause it just got really bad really quick . . . got pushed into walls, pushed into counters . . . [it was] emotional, verbal, very nasty, mean-mouthed. (Participant 3, autistic woman)*

*I didn’t realise that people deal with these things different than I would, I was kind of like ‘oh you’ve had a bit of crap growing up, you’ve gotten over it, you’re a functioning human being’ but obviously he was deeply, deeply affected by stuff and it came out with him not being the nicest person to me. (Participant 4, autistic woman)*

### Autism used against you

Just as some abusers used their own neurotype, mental health or life history as excuses for their behaviour, many found ways in which to exploit or manipulate the autistic victim based around being autistic.

#### Abusing through autistic traits

There was a clear pattern of abusers using the fact that the victim was autistic against them in some way to make the abuse more effective – many of them deliberately, as many participants had their diagnoses at the time they were in these relationships. Beyond the ways in which being autistic could be used to identify someone as a target for abuse ( ‘*I think I appeal to that kind of person, because I’ve always been a bit of a loner, not had someone to talk to, to tell’ Participant 29, autistic woman)*, participants specifically talked about their abusers deliberately triggering meltdowns, so that the abuser could then present the autistic victim as the ‘unreasonable one’:

*she played on my autism quite a lot . . . she would use stuff against me, she’d like trigger me to have a meltdown . . . then as soon as I had the meltdown she’d be like ‘see, you’re the problem, I have to put up with this’. (Participant 7, autistic woman)*

*I had the meltdown and he just said I’m being really emotional, really crazy. (Participant 14, autistic woman)*

#### Undermining the autistic victim

One of the classic abuse tactics mentioned above is gaslighting, and this was something which all our participants experienced. They talked about the fact that they felt particularly vulnerable to it, though, because they were autistic and were used to being the one who ‘got things wrong’:

*I thought I was misinterpreting myself . . . they’re saying this is enjoyable and this is fun, but I’m not liking it . . . in general in any kind of social situation I always got things wrong. (Participant 2, autistic non-binary person)*

*I grew up being told I was wrong, too sensitive, too serious, not ladylike etc . . . for a long time I wasn’t even sure who I was . . . being involved with domestic violence does that too. (Participant 28, autistic woman)*

By undermining the participant’s sense of self along with their sense of the relationship, the abusers were able to play on a lifetime of messages that the autistic people ‘get it wrong’ to perpetuate the abuse. The other way in which abusers often used the diagnosis to undermine autistic people was in the eyes of other people – making them out to be unstable, or unreliable narrators of their experiences, for example. By doing this, the abusers created distance between the autistic victims and those who could or should help them, making it harder for the victim to leave. This is an example of classic abuse tactic of isolation, but for our participants it often had the added factor of telling other people that the victim was autistic, playing on stereotypes about autistic people as being ‘bad at understanding other people/social situations’ to minimize or dismiss accounts of their abuse:

*[he] didn’t want me to see friends and family without his consent . . . he told them I was autistic and that I thought every little argument was abusive. (Participant 14, autistic woman)*

*she made me cut off all relationships I had with female friends . . . male friends it was more subtle . . . making it known she was disappointed I was going to see them, or talking about all the ways I was ‘bad at relationship’*, *when we did to undermine me. (Participant 15, autistic man)*

#### Misinterpreting harmful intentions

All our participants had been sexually assaulted and raped more than once, or had been in more than one abusive relationship. Many attributed this to not recognizing the signs of abusive behaviour, or of malicious intent in others:

*I trusted people to not do horrible things ‘cause I would never imagine that they would do those thing. (Participant 4, autistic woman)*

*he was the last in a line of people that kind of took advantage of me when I was younger . . . I didn’t really understand, you know, what really it would look like if somebody was interested in you . . . so technically [there] was consent, but not informed consent. (Participant 3, autistic woman)*

*I went straight out of that situation into an abusive relationship with my ex . . . that felt better, because I was like ‘yes, well she’s not doing these things to me, so it must be fine’’. (Participant 7, autistic woman)*

Participants emphasized that this was one areas where they felt that being autistic most strongly intersected with their experiences of victimization. This was because they assumed the best of other people, they struggled to believe people could want to do something other than what they literally said, or they felt that being autistic made them stand out as different, and therefore as a potential target:

*‘I did not read intentions well at all so I always felt sorry for him, I was like ‘oh there are all these reasons why he is being like this’ . . . he trapped me in his room, I couldn’t get out for the whole night. (Participant 7, autistic woman)*

*Now I look back and think how obvious it was right from the start that something wasn’t right . . . I take everything on face value, I am not good at reading between the lines. (Participant 28, autistic woman)*

Some participants felt that being autistic meant that they had difficulties understanding norms in relationships, and therefore they were vulnerable to abusive partners because they were less likely to challenge behaviours, even if they didn’t like them:

*He would have sex with me, whether I wanted to or not . . . part of it was my lack of understanding about how relationships work, and that my own feelings and wants and needs were as important. (Participant 29, autistic woman)*

One participant summed this up by saying:

*I suspect autistic women are less well-equipped . . . to identify people’s motivations, we’re less well-equipped to know what to do in response when someone makes us feel bad, because we’ve been made to feel bad since we were three years old. (Participant 17, autistic woman)*

### Poor family models

Many of our participants talked about the fact that they felt they had poor family models of relationships, and that this meant they were primed not to recognize abusive behaviour, or to misinterpret or excuse abusive behaviours, because it was the norm within their family:

*[my mother] will tell you that she got knocked up at 17 and married an abusive drunkard. (Participant 3, autistic woman)*

*mother [was] neglectful when we were very little, physically abusive, always threatening to put me in care. Father: physically and verbally abusive, and a gaslighting shit to this day!. (Participant 9, autistic woman)*

*my parents would not have passed an exam for being parents . . . my father’s idea of telling us we were bad was give us a backhander across the face, very fond of using pain . . . we were abused children and we accepted that was natural, well that’s how it happens. (Participant 12, autistic man)*

In the case of some participants, family members were their original abusers, adding another pattern to *‘Experiences of Abuse’*, from our earlier theme. This meant that from an (often incredibly) early age, sexual, physical and psychological abuse was part of their lives.

*my father used to molest me . . . I remember him doing it from the age of 6 or so, but I don’t remember when it started. He used to put his hands down inside my underwear, I used to roll away but he kept coming back. (Participant 2, autistic non-binary person)*

*when I was only three I was sexually abused by my male paternal cousin. (Participant 9, autistic woman)*

This obviously has nothing to do with the participant being autistic and is entirely to do with the abuser – which is the case in any situation where someone chooses to abuse another person – but it is worth recognizing that being autistic can be a risk factor for abuse in childhood, especially when there are co-occurring cognitive or language delays. Our participants did not report having these delays, but their childhood experiences had a long-lasting impact as they set a pattern for what they thought were normal interactions:

*I actually have always ended up with quite exploitative relationships, honestly. (Participant 18, autistic woman)*

The other way in which experiences with families, and at school, were important was around learning about sex and relationships. Most participants, with a couple of notable exceptions, had no conversations with their parents about what to expect from sex or romantic relationships, which they felt left them unprepared for the reality of this aspect of life:

*from my parents, no – like we didn’t talk about it at all, very repressed family, pretty sure my parents are both on the spectrum somewhere (Participant 4, autistic woman)*

*the parents kind of all assumed that the school would sort everything out, and the school always assumed that the parents would sort everything out. (Participant 17, autistic woman)*

### Impact of/on friendships

Along with family relationships, friendships were repeatedly mentioned by our participants as being important in their experiences of abuse. Some had friends who were themselves manipulative or abusive:

*an old best friend when we were about 12, we’d gone to the swimming pool together and afterwards we were in the changing rooms . . . .he threw my clothes and my swimmers which I’d taken off into his cubicle . . . he wanted me to do everything. (Participant 2, autistic non-binary person)*

*she had bullied me before . . . I pretended to be friends with her for an easier life. (Participant 9, autistic woman)*

Some participants also talked about the fact that they deliberately mimicked certain peers or friends who they thought were socially successful, and that this led them into behaviours which they would not have necessarily chosen otherwise:

*one of my only friends, basically, that I had for a long time . . . she is the one that talked me into basically trying [sexual] things with the boyfriend at the time, dating older guys who were not really appropriate. (Participant 3, autistic woman)*

This kind of masking was something which several participants had experienced. They did not always feel that this contributed to abuse in their lives, but that they did end up behaving in line with their ‘masked persona’ rather than their authentic selves, which impacted relationships:

*over time I became the funniest guy in the group which gave me acceptance, a very false acceptance . . . I land up sleeping with a girl that I had absolutely no emotional feelings for or even sexual feelings for. (Participant 12, autistic man)*

Both in regards to friendships and romantic relationships, participants talked about the impact of unmasking as much as the impact of masking. Often, when they were unable to maintain their masked persona for some reason, or when they chose to behave in a more authentic way, this was received negatively by those around them:

*after I got pregnant and the hormones kicked in, I had zero spoons [energy] for anything else. I couldn’t mask anymore . . . he found that difficult and so things got worse. (Participant 28, autistic woman)*

*after [the rape] I really struggled to keep up my mask in big groups and social situations, especially parties like where it happened . . . some of my friends kind of dropped me and stopped inviting me because ‘I wasn’t like before’. (Participant 4, autistic woman)*

#### Lack of protective friendships

For most participants, though, the main thing they focussed on was a lack of friendships across their lives, which meant that they missed out on the protective advice or intervention which many non-autistic people benefit from. For example, they had no-one to talk to about it when they were abused, or to check whether what they were going through was normal:

*I didn’t have that girl group that would normally be like ‘no dude, don’t get involved with that guy’ because those girl groups, in my case, they weren’t around. (Participant 3, autistic woman)*

This meant that our autistic participants were relatively isolated even before their abusers started to cut them off from the few friends and connections they had. They were therefore more reliant on their abusive partner as their main relationship than many non-autistic people would be, again making it harder to leave or challenge the behaviours.

*I used to rely all on one person, that’s one thing you shouldn’t do . . . whoever you’re with. (Participant 22, autistic woman)*

#### Losing friends due to disclosure

Another aspect of interactions with friends which several participants discussed was their friends’ reactions to being told about the abuse the autistic person had experienced. While some had supportive friends, or family members, many had much more negative reactions from those around them. For example, some had friends who denied that the assault or abuse had happened, or who ended the friendship over the disclosure:

*my friends . . . hadn’t believed me – like my best friend, who kept saying ‘Oh, I just think it’s the dynamic between you two’. (Participant 17, autistic woman)*

*I tried to tell a friend, whose friend it was that [raped me], on the first occasion, but it didn’t go well . . . I lost a friend because of it . . . she didn’t want to deal with it, it was better to pretend it didn’t happen, or didn’t happen how I was saying it happened. (Participant 4, autistic woman)*

Overall, the lack of social support autistic victims felt was available to them had a significant impact on their ability to recognize abusive behaviour, and then to seek help with addressing that abuse. Many felt that they were left to manage their experiences and their trauma alone, which leads into the next theme.

### Handling trauma

All our participants recognized that the experiences they had been through would generally be considered traumatic. For some, this had the same kind of impact as expected on non-autistic people, such as feeling anger or developing PTSD:

*I was absolutely ragingly angry with everybody . . . it’s not their fault but it just really had an impact on our relationships. (Participant 18, autistic woman)*

*I had to have quite extensive therapy for PTSD. (Participant 20, autistic woman)*

However, many said that they felt the events did not hold an ongoing power over them, instead minimizing the impact or seeing it as a separate or inevitable part of life:

*I did what some [autistic] people tend to do, we compartmentalise it, so I just put it away for a while. (Participant 22, autistic woman)*

*there’s a sort of general acceptance because it’s so horrifying, you’re so powerless to do anything about it, so you have to accept it on some level . . . it’s almost like you’re in trauma or it’s over, it’s just black and white. (Participant 25, autistic woman)*

This response appears to be trying to neutralize the traumatic experience as part of someone’s biographical sense of self. Some participants put this down to their ability to remove emotion from how they reacted or thought about something, which they felt was a strength of being autistic in these difficult situations:

*I approach decisions with an excruciating level of rationality and logic . . . it’s mostly been quite helpful, I’ve taken a very practical view on everything. (Participant 15, autistic man)*

#### Shutting down

Most of our participants recalled that as things were happening to them, their automatic response was to shut down, and try to shut it out, rather than to react aggressively or even by trying to run away:

*I think that predisposed me to . . . I was less likely to put up resistance and more likely to blame myself. (Participant 20, autistic woman)*

*I was very, very passive . . . I’d just float along, a situation would happen to me. (Participant 22, autistic woman)*

Many attributed this to ‘*passivity*’ – both in terms of things being done to them, and in terms of appearing as someone whom things could be done to. Because of this passivity, which was often a learned response to a lifetime of being told that their natural responses were wrong both at home and in school, many victims were then reluctant to report what had happened to them. Many felt that they wouldn’t be believed, because they hadn’t said ‘no’, or thought that they had not said it ‘the right way’:

*I didn’t tell anyone! I just thought I’d get in trouble . . . I just didn’t think I’d be believed, to be honest. (Participant 22, autistic woman)*

Autistic people also explicitly talked about blaming themselves for what had happened based on their response, or lack of response, and their perceived difficulties in interpreting social interactions:

*At the time I felt like, I’d definitely done something wrong and I was very worried about getting him into trouble, because I didn’t trust myself to know I had read the situation right. (Participant 7, autistic woman)*

#### Long-term impact on relationships

Understandably, while some participants felt that there had been minimal impact from their abuse, the majority said that these experiences had significantly changed how they approached relationships. For some, this impact was to help them make better and safer relationship choices, because it showed them what signs to look out for, and they knew that their current relationships were healthy because they did not have the same behaviours as previous ones:

*there was a couple of times when I was like ‘oh, this is a red flag for me’, and we’d talk it over . . . I’d be worried if she didn’t want to talk. (Participant 7, autistic woman)*

*I’ve developed skills to identify my boundaries and the kind of boundaries I don’t want people to cross . . . I trust him, I don’t think he’s going to be scheming for ways to manipulate me. (Participant 20, autistic woman)*

A minority of participants, though, found their experiences had the opposite effect. Rather than giving them an indication of what good relationships looked like, their experiences left them scared and suspicious of anyone who sought out a relationship with them:

*I used to assume the best intentions, and now I just go ahead and assume the worse. (Participant 3, autistic woman)*

*I avoid people in general as much as humanly possible. (Participant 4, autistic woman)*

*I see the vast majority of men as potential sexual predators, whereas a neurotypical person would think ‘ok, this has happened but not all men are like that’. (Participant 9, autistic woman)*

A minority took this to the point of extrapolating from how they had been treated by one person to ‘all people are like that really, so I might as well do what I want’, and becoming very cold and transactional in how they talked about sexual partners:

*I’ve found for myself many time the trade-off is sex . . . if I sexually satisfy you . . . .you can tell me about normal life. (Participant 12, autistic man)*

It is not surprising that a range of different people would have a variety of long-term responses to repeated victimization. This variation highlights the need for better understanding of these experiences among autistic people, and support for those who have been victimized, to try to ensure that future relationships are positive rather than leading to further isolation.

### Recommendations for relationship and sex education (RSE)

We also asked our participants what they wished they had been taught earlier in life, considering that they had generally had very little RSE. Along with the lack of open and honest discussions about sex and relationships mentioned in *Poor family models*, the RSE our participants had received at school was felt to be woefully inadequate. Some had no RSE, due to their age or location, and most had classes which focussed solely on the biological and physical mechanics of the process, with no discussions of healthy relationships or consent:

*I think we just had some kind of basic biological teaching . . . as if it was the 1950’s. (Participant 25, autistic woman)*

*it was more about biology than anything else, nothing about relationships. (Participant 27, autistic woman)*

Most participants said they wished that they had been taught about healthy relationships, not just about the physical acts of sex and pregnancy; about consent; and for education to be more gender balanced:

*[they need] this is what it looks like in a good relationship, this is what consent actually looks like out in the real world. (Participant 3, autistic woman)*

*you’re not equipped to know how do you tactfully, or even un-tactfully, just say, well, no . . . they’ve ended up doing things that they didn’t really want to do because they just didn’t know how to say no. (Participant 17, autistic woman)*

*I think, it’s kind of quite easy to be the stereotype of the boy pushing for things . . . but I think the [idea that women can be abusive], helping to recognise that that’s not healthy as well, that would be useful. (Participant 15, autistic man)*

This highlights the significant gaps in what young autistic people have generally been taught about sex and relationships, as it cannot be assumed that they will get information from their peers in the same way as non-autistic teenagers do. Indeed, one 25-year-old participant highlighted that she had almost entirely learned about sex from pornography, and that while she knew this was not necessarily realistic, she had no other models of what to expect, so she planned to avoid sex altogether:

*I wouldn’t want to have sex with anyone . . . I genuinely don’t want to. (Participant 8, autistic woman)*

While our participants covered a wide age range and therefore a variety of educational experiences, that even younger participants felt so unprepared was concerning. It suggests that the patterns of negative experiences related by our participants may still take some time to challenge and break, if education is not supporting younger autistic people to develop healthy relationships.

## Discussion

This study presents a qualitative exploration of autistic people’s experiences of IPV, SA and domestic abuse. Many of the incidents and themes described reflected classic abuse tactics and experiences, but participants discussed the ways in which they felt being autistic had intersected with their relationships to mark them out as potential targets, or made it harder to recognize and respond to the abuse. They also discussed the lack of education they received, which meant they were unprepared for relationships, and the kind of things they would have liked to have learned to equip them better.

The findings in this article, that autistic people have often been multiply victimized, either through repeated assaults or multiple abusive relationships, directly echoes the findings from larger-scale interview–based work with autistic people (Pearson et al., 2023). That study also found that participants had multiple experiences of victimization, though they were exploring a broader range of types of victimization, such as including mate crime and difficult employment situations. While this study specifically recruited people who had experienced IPV or SA, the extent, severity and frequency of childhood abuse was greater than would be expected in a non-autistic group of people, and is in line with other work about the prevalence and age of first instance among autistic people (Cazalis et al., 2022). Taken together, this study and previous work reinforce that experiences of abuse are incredibly common among autistic people.

This study sought to build on that knowledge by exploring how autistic people felt that being autistic had interacted with or contributed to their experiences of abuse. This is not to apportion blame to the autistic individuals, because it is always the perpetrator’s choice to abuse, but to understand how being autistic may shape these experiences and how people handle them. Most participants felt that being autistic had contributed in some way, often saying that they felt they presented an easier target for abusers, because they were more trusting, found it harder to communicate a lack of consent or distress, or because they lacked the social support to recognize abusive behaviour and then leave. This supports previous work with autistic women, who talked about struggling to recognize malicious intent in others or to ‘read between the lines’ when being propositioned, and how being autistic meant that they found it harder to identify whether something had been SA or abuse (Sedgewick, Crane, et al., 2019). Similarly, research has recognized that especially for autistic children and young people who have cognitive and/or language delays, these factors may embolden abusers or influence which child they decide to victimize (McDonnell et al., 2019; Pfeffer, 2016). It is likely that, even though our participants were mostly verbal and did not have co-occurring intellectual disability, aspects of being autistic made them stand out to perpetrators in similar ways.

One of the novel findings of this work is the descriptions of ways in which abusers deliberately ‘weaponised’ features of autism against the participants. The most commonly discussed were, first, playing on the victims history of difficulty in social situations to reinforce and strengthen gaslighting tactics – emphasizing the message that they didn’t understand social interactions properly to pretend that behaviours weren’t abusive. Second, by deliberately triggering meltdowns (an abusive behaviour in itself), and then using that to present the autistic victim as overly emotional, irrational and potentially even dangerous, both to themselves and to people outside the relationship. This acted to undermine the autistic person if they did tell someone what was happening or try to reach out for help, because people in their lives were predisposed to think that the autistic person was an unreliable narrator of events. Participants described this being the case with friends, church communities and even family members, and how these relationships sometimes suffered after they had left their abuser because those people felt it was the victims’ fault in some way. While this is not unusual for autistic or non-autistic survivors, but where our participants and their abusers knew that they were autistic at the time of the abuse (often the case in adult romantic relationships), the abuser had often specifically played into stereotypes about autistic people’s social capacity to convince other people that the participant had interpreted the situation wrongly.

Using autistic features to harm an autistic person is stigma and prejudice in action, and could be classified as a hate crime. Experiences of aggression, and oppression, based on being autistic, have been explored by autistic scholars through the lens of Minority Stress Theory (Meyer, 1995). Minority Stress Theory argues that people with minority or marginalized identities experience more stressors in their everyday lives, and that this contributes to poor mental health and overall outcomes. Autistic people are a neurominority, being neurodivergent in a majority neurotypical world, and therefore can experience identity-based discrimination in the same way as minority ethnic or sexuality groups can, with similar negative impacts on their mental health (Botha & Frost, 2020). In terms of autistic experiences of abuse, Pearson et al. (2023) explicitly placed their research within Frost’s model of social stigma and its consequences (Frost, 2011). Through this lens, they argued that abuse should be seen as a form of ‘situational’ vulnerability rather than personal vulnerability – that being in a situation where the other person is willing to manipulate features of your neurotype to harm you is not something which should be seen as the victim’s fault. This was echoed in interviews with our participants, who talked about the differences between the unhealthy and healthy relationships they had been in, and how in good relationships, their partners had acknowledged the differences which came with their neurotype, but did not use these to harm or manipulate them as abusers had.

That several of our participants discussed ways in which patterns of abuse had been present in their lives even from childhood was an unexpected finding. Research with non-autistic people has previously shown that children who grow up in households where domestic or sexual abuse is present are more likely to be in abusive relationships as adults. This appears to apply to autistic people as well, and may be especially important in making it hard for them to identify healthy and unhealthy relationship behaviours from others, because they are likely to have fewer alternative models for social interactions outside the family, due to their social differences and often social isolation. As autistic children whose parents report adverse childhood experiences (ACEs) are themselves more likely to have ACEs (Andrzejewski et al., 2023), it is likely that a similar inter-generational pattern can occur around abusive relationships.

Another important finding in this study was the variety of ways in which autistic people described responding to having been assaulted or in abusive situations. Some were very matter-of-fact about their experiences, and felt that they had moved on by drawing clear boundaries around it. This type of compartmentalization or minimization could be seen as a parallel to the dissociation which is common as a response to trauma – one which often interferes with healing from the experiences. It has been shown to be a common experience among autistic adults who have experienced interpersonal trauma (such as SA or IPV) (Reuben et al., 2021). That this is such a widespread response among autistic people who have been through these kinds of trauma is important for authorities and support providers to know, as it will influence the ways in which they work with survivors.

Other participants discussed how there had been long-term impacts on their romantic and sexual relationships, from helping identify healthy and positive behaviours in a partner to a generalized suspicion (particularly of cisgendered men) and reluctance to engage in new partnerships. Our participants also talked about having developed PTSD in response to their experiences. PTSD is a common response to having been a victim of IPV (Dutton et al., 2006), and it is not surprising that this was also the case in this sample. As mentioned previously, autistic people are more likely to have PTSD than non-autistic people, especially around social incidents (Haruvi-Lamdan et al., 2020; Rumball et al., 2020, 2021), and this study emphasizes that message. It is possible that for autistic people, who are often already socially marginalized, the impact of relationship-based abuse is more intense than for non-autistic people, and therefore may contribute more to the development of PTSD. More work needs to be done to understand the ways in which PTSD among autistic people based on IPV and SA develops and is experienced, so that more effective support can be designed to help this particularly vulnerable group. That several participants talked about their experiences in ways which might appear unusual to professionals who are not knowledgeable about autism could become a barrier to accessing support, as has been seen in physical health settings (Doherty et al., 2022), and policies for working with neurodivergent victims are needed to improve care and potential prosecution rates.

Another form of support which was lacking in the experience of our participants was adequate RSE. Many had only received basic instruction in the biology and mechanics of sex, with no discussion of consent or good versus abusive behaviours, meaning that they were, in the words of one participant, ‘ill-equipped’, for the reality and nuance of adult relationships. This lack of high-quality and wide-ranging RSE for autistic young people has been discussed in previous literature, highlighting that many are not taught what are appropriate behaviours either for them to engage in or to receive from others (Beddows & Brooks, 2016; Hannah & Stagg, 2016). This has been shown to be a factor in vulnerability to SA and IPV for autistic people of all genders in adulthood (Brown-Lavoie et al., 2014). Even our younger participants had struggled to access good RSE, despite being of an age where RSE was mandatory during their school years, instead often finding out information themselves from less reputable sources, and this left them with potentially harmful models of what to expect in a sexual relationship. It is also worth noting that none of our participants explicitly mentioned that they had attended anything other than mainstream schooling, and so autistic people in special educations settings may receive even less effective RSE, where it may be assumed that young people do not ‘need’ it due to stereotypes about their likelihood of engagement in romantic or sexual relationships (Sedgewick, Crane, et al., 2019). While there is relatively little work on RSE for autistic young people, studies consistently show that this group feel they would benefit from more, and more tailored, RSE (Cheak-Zamora et al., 2019; Hannah & Stagg, 2016). Considering that all children and young people in the United Kingdom now have to receive some form of RSE as part of their compulsory schooling, this seems like an obvious target for improving the information given to young people of all neurotypes about healthy relationships. Doing so may help to prevent future instances of autistic people being victimized, and the accompanying long-term mental health consequences.

The research team and advisors were neurodiverse with varied lived experiences of sexual victimisation (SV) and IPV. These personal perspectives on the issue have the potential to introduce subjectivity, as with any form of ‘insider research’. However, the range of experiences discussed by participants did not directly match with those of the study team, giving some distance and reducing the likelihood of warping the interpretation. The separate initial coding of the data also ensured independence and gave space for discussion about where authors may have brought their personal views into their codes. We would argue that the make-up of the team was a strength of this work, and ensuring the input of people with lived experience is valuable in any study in this area. It meant that the measures could be designed collaboratively and phrased in ways which were likely to be the least distressing for participants, for example. Most importantly, it meant that participants felt they could trust that researchers would understand what they were talking about and were therefore more able to be open and share the truth of their experiences – incredibly valuable in qualitative research on sensitive topics.

### Limitations

As with all research, there were limitations to this project. First, the relatively small sample size means that the findings should not be over-generalized. This is not unusual for qualitative work, and the representation of autistic people of many genders is a strength of the sample, although this would ideally be more equally balanced to ensure exploration of the abuse experiences of men and non-binary people who are often overlooked in victimization research. Second, we recognize that the sample is highly uniform in being majority White, late diagnosed and mostly using verbal communication. It is likely that autistic people of colour, who are multiply marginalized, will have somewhat different experiences of abusive relationships, and of seeking formal support, which are not included in this study. Those who are diagnosed later in life are likely to have different vulnerabilities and experiences than those who have an earlier diagnosis, which often accompanies more obvious support needs. This is especially the case for those who may rely more on caregivers, or be less able to communicate things that have happened to them, particularly in childhood. Equally, those who use non-verbal communication will have different experiences and challenges around the topic which would be valuable to further research. We did not collect information about sexual orientation from all participants, although participants discussed both different- and same-gender experiences – future research should do so as non-heterosexual people may be more likely to experience interpersonal victimization, and less likely to seek support (Rothman et al., 2011; Tillewein et al., 2023). It is also worth noting that it was beyond the scope of this study to evaluate the impact of co-occurring neurodivergences or mental health diagnoses on autistic people’s experiences of victimization, something which future research should investigate. Finally, this study did not focus specifically on experiences of interacting with services, which would be a crucial issue to understand in more depth to create recommendations for best practice in supporting autistic abuse victims.

## Conclusion

This is one of the first studies to interview a wide range of autistic adults about their experiences with IPV, SA and domestic abuse, and to ask for their reflections on how this interacts with their diagnostic status. We found that many autistic people’s experiences were in line with what is known about general patterns of abuse, such as the tactics used to perpetuate it. However, we also showed that there are important ways in which being autistic changes or shapes these experiences, in terms of being targeted, struggling to recognize the abuse and their abuser using autism against them in various ways. Developing better RSE for autistic young people in school, and supporting families to talk about safe and healthy relationships at home, would empower autistic people to recognize and respond to abusive behaviour from the first instance, and potentially to avoid people who display warning signs altogether. Similarly, working with autistic people who have experienced abuse to design and implement training for social workers, police and survivor’s charities could go some way towards improving the support available. It is important for families, schools and support workers to understand how autistic people may experience abuse differently to non-autistic people, so that better systems can be created to catch these instances earlier and put a stop to them, and to help autistic survivors recover from their experiences as positively as possible.

## Supplemental Material

sj-docx-1-aut-10.1177_13623613231205630 – Supplemental material for Experiences of interpersonal victimization and abuse among autistic people Supplemental material, sj-docx-1-aut-10.1177_13623613231205630 for Experiences of interpersonal victimization and abuse among autistic people by Sarah Douglas and Felicity Sedgewick in Autism
