# Supplementary material for: Experiences of interpersonal victimization and abuse among autistic people
Source: Autism. 2023 Oct 16;28(7):1732–45. doi: 10.1177/13623613231205630 (PMC11191373; doi:10.1177/13623613231205630)
Supplement: sj-docx-1-aut-10.1177_13623613231205630 – Supplemental material for Experiences of interpersonal victimization and abuse among autistic people [file sj-docx-1-aut-10.1177_13623613231205630.docx]

Sexual Victimisation Interview Schedule

You’ve got in touch with me and said that you want to take part in this research because you have some personal experience of sexual or domestic violence. Could we start by you telling me a bit about those experiences?

- What happened?
- How old were you?
- Was it someone you knew or a stranger?
- Did you trust them, or believe they knew best?
- How did you feel about it at the time?
- Was it something you thought you wanted to happen?
- Did you tell anyone at the time?
- Have you told anyone since?
- Is this the only time you have experienced sexual or domestic abuse?
- What else happened?
- How did these experiences tie in to your romantic and sexual relationships more generally?
- Do you think they had a lasting impact on you and your relationships? In what ways?
- Some people have said that being autistic contributed to misinterpreting situations or not trusting their instincts about someone. Do you think being autistic had any role in what happened your case?
- Do you think there are any patterns to what has happened to you?
- Is there anything you do now to avoid future bad experiences? For example, some people get friends to meet and vet anyone who they are interested in a romantic relationship with.

I’m also interested in your experiences of sexual and relationship education, because I’m trying to understand what autistic people were taught and how we might improve this in the future.

- What kind of sexual/relationship education did you have when you were a teenager?
- Do you think this prepared you for sex or for romantic relationships?
- Did you pick up information from any other sources than formal education? What were these sources? What information did they give you?
- What else do you wish you had been told?
- Was there anything important that you think was missed?
- Would it have been more useful if it was taught a different way? What do you think would have worked best for you?

Thank you for talking to me about these difficult experiences, I really appreciate it and will treat all your data with respect. Is there anything else you would like to tell me?
